# Achieving Glycemic Targets in and out‐of‐School: Real‐World Data From 1341 Italian Children Using the MiniMed 780G System During Auto Mode

**DOI:** 10.1155/pedi/1653728

**Published:** 2026-04-08

**Authors:** Jen McVean, Riccardo Schiaffini, Marta Bassi, Barbara Piccini, Benedikt Voelker, Vittorino Smaniotto, Tim van den Heuvel, Ohad Cohen

**Affiliations:** ^1^ Diabetes Operating Unit, Medtronic, Tolochenaz, Switzerland; ^2^ Diabetes Unit, Bambino Gesù Children’s Hospital, Rome, Italy, ospedalebambinogesu.it; ^3^ Department of Neuroscience, Rehabilitation, Ophthalmology, Genetics, Maternal and Child Health, University of Genoa, Genoa, Italy, unige.it; ^4^ Pediatric Clinic, IRCCS Istituto Giannina Gaslini, Genoa, Italy, gaslini.org; ^5^ Diabetology Unit, Meyer Children’s Hospital IRCCS, Florence, Italy

## Abstract

In this real‐world analysis, we evaluated glycemia and insulin delivery in Italian children using the MiniMed 780G system during auto mode use, comparing days with a school routine to out‐of‐school (OOS) days. Data from 1341 users, self‐reported under 16 years old and with type 1 diabetes (T1D), showed no meaningful difference in time in range (TIR) between school days (73.4%) and OOS days (72.4%), and international targets were met on average during both types of day. Minor sensor glucose variations were observed during school hours, such as a more pronounced glucose peak after breakfast and a clearer dip before lunch on school days. The insulin delivery algorithm effectively managed these fluctuations. Maintaining optimized glycemia during both school and OOS days may enhance learning and support cognitive and brain development.

## 1. Introduction

The importance of establishing and maintaining as close to normal glucose levels as soon as possible to avoid short‐ and long‐term complications is well established in people with type 1 diabetes (T1D) [1]. This is especially important in children who will live with T1D for most of their lives. Hyperglycemia is harmful not only in the long term, but also at a young age as it negatively impacts brain development and cognitive function in children [2]. As children spend roughly one third of their weekdays in school, ensuring stringent glucose control during school days (as well as during other times) is paramount to the academic success and health of children living with T1D (CwT1D).

Children have differences in sleep schedules, food intake, and physical activity on school days versus weekends and holidays. Juggling this variability in schedules may be challenging for glycemic control in CwT1D and their caregivers. Data have been mixed regarding whether glycemia is better, worse or the same on school days versus non‐school days, in the pre‐automated insulin delivery era. A study published in 2016 demonstrated that hemoglobin A1c’s in children in the US were equivalent during summer break versus the school year and quite elevated at 8.5% [3]. Another study published in 2023 found that time in range (TIR) was higher during daytime hours (8:00–15:00 h) on weekends (54%) than on school days (51%). TIR was well below the international recommendation of 70% on school days and weekends [4].

With the recent advances in the automation of insulin delivery and the second‐generation MiniMed 780G system (which delivers auto basal as well as auto correction insulin every 5 min as needed during auto mode), we hypothesized that children using this system in auto mode could achieve consistent glycemic control on school days as well as out‐of‐school (OOS) days (i.e., weekends and holidays). Importantly, we also hypothesized they would meet or exceed the international targets for TIR. In this real‐world analysis, we aimed to evaluate the glycemia and insulin delivered in Italian CwT1D using the MiniMed 780G system in auto mode on days with a school routine compared to days without.

## 2. Methods

This study is a retrospective, real‐world analysis using data from CareLink Personal, the manufacturer’s software platform that allows MiniMed users and their healthcare professionals to generate reports and monitor diabetes management progress. Data from users of the MiniMed 780G system was extracted and analyzed using a validated methodology as described by Van den Heuvel et al. [5], which has also been applied in other real‐world analyses of CareLink Personal. Regarding the representativeness and data quality of this database, over 95% of MiniMed 780G system users had a CareLink Personal account (in 2023). In the Europe, Middle East, and Africa region, 94% of users (or parents/guardians) had provided informed consent for their data to be used in research, and 99% of all data uploads were performed automatically on a nightly basis (Italy‐specific data was not available). The study’s observation period spanned from January 1, 2024 to December 31, 2024. Approval from an ethics committee was not required per Italian legislation.

To be eligible for the study, MiniMed 780G system users needed to have a registered CareLink Personal account in Italy, self‐report as having T1D and being under 16 years old and have parental (or legal guardian) consent provided. Additionally, users were required to have at least 2880 continuous glucose monitoring (CGM) data points, an equivalent of 10 days of data, during mornings on school days and OOS days [6]. Only days in which 100% of the time was spent in auto mode were included, as we were specifically interested in the performance of the algorithm itself and avoiding interference from manual use, and wanted to ensure a uniform system use across school and OOS days.

The endpoints centered on metrics related to glycemic control, carbohydrate announcements, insulin delivery, and system settings, with further details provided in Table 1. The analysis was descriptive, aggregating data at a 24‐h level for school days and OOS days. The 2024 holiday schedule was determined based on definitions provided by the Italian Ministry of Education. Sub‐analyses were conducted for data aggregation on an hourly basis as well as within the defined “school window” (8:30–13:30 h. Finally, a sensitivity analysis was conducted, mirroring the main analysis but without the 100% auto mode criterion.

**Table 1:** Continuous glucose monitoring based metrics of school days versus out‐of‐school days.

Metric	24 h window		School window (8:30–13:30 h)	
	School days	OOS days	School days	OOS days
General				
Users count	1341	1341	1341	1341
System use (days)	137	147	137	147
Sensor use (%)	98.5	98.3	98.5	98.3
Auto mode use (%)	100	100	100	100
Glycemic metrics				
SG mean (mg/dL)	150.0 ± 12.7	152.0 ± 12.8	148.0 ± 17.0	140.0 ± 13.9
SG std (mg/dL)	51.1 ± 9.1	53.1 ± 9.4	47.9 ± 10.3	47.3 ± 10.3
SG CV (%)	33.9 ± 4.5	34.9 ± 4.5	32.1 ± 5.3	33.6 ± 5.4
GMI (%)	6.9 ± 0.3	6.9 ± 0.3	6.9 ± 0.4	6.7 ± 0.3
TIR (%)	73.4 ± 8.5	72.4 ± 8.4	74.1 ± 11.8	79.0 ± 9.5
TITR (%)	48.0 ± 8.7	48.2 ± 8.4	48.4 ± 12.7	58.0 ± 11.4
TB70 (%)	2.2 ± 1.7	2.0 ± 1.6	2.4 ± 2.4	2.4 ± 2.3
TB54 (%)	0.4 ± 0.5	0.4 ± 0.4	0.4 ± 0.64	0.4 ± 0.6
TA180 (%)	24.5 ± 8.6	25.7 ± 8.5	23.5 ± 11.9	18.5 ± 9.3
TA250 (%)	5.0 ± 3.9	5.9 ± 4.2	4.4 ± 4.9	3.7 ± 3.6
Carbohydrate announcement and insulin delivery				
TDD (units)	41.8 ± 21.0	41.3 ± 20.9	8.6 ± 4.3	9.6 ± 4.8
Auto basal dose (%)	41.2 ± 5.5	40.7 ± 5.3	39.9 ± 9.9	35.3 ± 10.0
Auto correction bolus dose (%)	21.4 ± 6.4	21.5 ± 6.5	20.8 ± 8.6	14.7 ± 7.0
User‐initiated bolus dose (%)	37.4 ± 9.9	37.8 ± 9.9	39.3 ± 15.1	50.0 ± 14.6
User‐initiated boluses per day	5.2 ± 1.7	5.2 ± 1.9	1.3 ± 0.6	1.5 ± 0.6
Carbs entries per day	5.1 ± 1.7	5.1 ± 1.8	1.3 ± 0.6	1.6 ± 0.6
Carbs per day (g)	189.0 ± 66.4	190.0 ± 70.1	44.3 ± 23.3	60.9 ± 27.2
Carb ratio mean	15.1 ± 8.4	15.3 ± 8.5	15.3 ± 8.9	14.8 ± 8.4
Distinct carb ratios per day	2.7 ± 1.0	2.6 ± 0.9	1.0 ± 0.4	1.2 ± 0.4
Alarms per day	9.2 ± 5.5	9.4 ± 5.5	1.9 ± 1.4	2.0 ± 1.47
System settings				
Time with GT 100 mg/dL (%)	47.0 ± 44.8	47.8 ± 45.5	47.0 ± 44.9	47.7 ± 45.4
Time with GT 110 mg/dL (%)	26.1 ± 36.7	25.9 ± 37.5	26.1 ± 36.7	25.9 ± 37.5
Time with GT120 mg/dL (%)	24.4 ± 37.7	24.1 ± 38.1	24.3 ± 37.6	24.1 ± 38.1
Time with temp target (%)	2.2 ± 5.5	1.9 ± 5.2	2.2 ± 6.5	2.0 ± 5.2
Time with AIT 2 h (%)	55.2 ± 45.2	56.2 ± 45.9	55.2 ± 45.2	56.2 ± 45.9
Time with AIT 2–3 h (%)	42.1 ± 44.5	41.2 ± 45.2	42.1 ± 44.5	41.2 ± 45.2
Time with AIT 3–4 h (%)	2.7 ± 13.7	2.6 ± 14.0	2.67 ± 13.7	2.6 ± 14.0
Time with AIT 4 h (%)	0.0 ± 1.0	0.0 ± 0.6	0.0 ± 1.0	0.0 ± 0.6

*Note:* Data are shown as means ± standard deviation.

Abbreviations: AIT, active insulin time; CV, coefficient of variation; GMI, glucose management indicator; GT, glucose target; OOS, out‐of‐school; SG, sensor glucose; std, standard deviation; TA180, time above 180; TA250, time above 250; TB54, time below 54; TB70, time below 70; TDD, total daily dose; TIR, time in range; TITR, time in tight range.

## 3. Results

**Figure 1:**
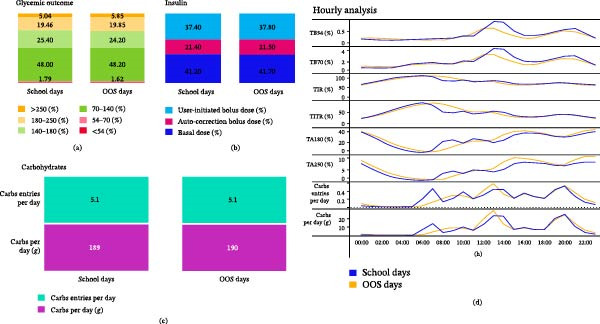
Continuous glucose monitoring based metrics of school days versus out‐of‐school days. Subfigure (a) shows time in ranges, aggregated over 24‐h. Subfigure (b) shows distribution of insulin delivery, also aggregated over 24‐h. (c) Number of carbohydrate entries and grams per day, similarly aggregated over 24‐h. Subfigure (d) shows data aggregated at an hourly level. Data are shown as means. TA180, time above 180; TA250, time above 250; TB54, time below 54; TB70, time below 70; TIR, time in range; TITR, time in tight range.

In total, 1341 MiniMed 780G system users were included. On average, they spent 284 days on the system, of which 137 were school days and 147 were OOS days. Figure 1a shows the endpoints for 24‐h level. On school days, TIR was 73.4 ± 8.5 and time below range (TBR) was 2.2 ± 1.7%. On OOS days, TIR was 72.4 ± 8.4% and TBR was 2.0 ± 1.6%. There were on average 5.1 ± 1.7 carbohydrate entries and 189.0 ± 66.4 g of carbohydrate entered on school days and 5.1 ± 1.8 carbohydrate entries and 190.0 ± 70.1 g of carbohydrate entered on OOS days. The average total daily insulin delivery (TDD) on school days was 41.8 ± 21.0 units, with 41.2% ± 5.5% delivered via auto basal, 21.4% ± 6.4% via auto correction, and 37.4% ± 9.9% manually. On OOS days, the TDD averaged 41.3 ± 20.9 units, with corresponding delivery percentages of 40.7% ± 5.3% for auto basal, 21.5% ± 6.5% for auto correction, and 37.8% ± 9.9% for manual delivery. There were no relevant differences in terms of glucose target (GT) setting and active insulin times (AIT) setting between school and OOS days. For details on this as well as additional information on glycemic control, carbohydrate announcement, and insulin delivery, we refer to Table 1.

Figure 1b illustrates hourly data, highlighting two key observations. First, during school hours (8:30–13:30 h) the patterns of carbohydrate announcements differ between school and OOS days, but they align after school. On school days, there are distinct spikes in carbohydrate announcements around 7:00 AM (breakfast) and 10:00 AM (school break), whereas this is not prevalent during OOS days. Second, post‐prandial hyperglycemia following breakfast is more noticeable on school days compared to OOS days, with a more prominent glucose dip occurring just before lunch. On the contrary, time above range (TAR) after school and during early evening seems more prominent during OOS days. During school days, the “school window” TIR was 74.1% ± 11.8%, and “school window” TBR was 2.4% ± 2.4%. On OOS days, this was 79.0% ± 9.5% and 2.4% ± 2.3%, respectively.

Results from the sensitivity analysis can be found in the supplement. The main analysis includes the 100% criterion and is based on 284 days. This is 90% of the total number of days from the sensitivity analysis (317 days).

## 4. Discussion

This study is the first large‐scale real‐world analysis of glycemic control in CwT1D in relation to school, using the MiniMed 780G system in auto mode. When comparing school days to OOS days at a 24‐h level, glycemic control was found to be similar. TIR was 73.4% on school days and 72.4% on OOS days, while TBR was 2.2% and 2.0%, respectively—both surpassing international criteria. Furthermore, no meaningful differences were observed in carbohydrate announcements, insulin delivery, or system settings between school and OOS days.

When examining the school window specifically (8:30–13:30 h), it became clear that school days exhibit distinct and more structured behavior patterns compared to OOS days. On school days, CwT1D showed a clear breakfast routine around 7:00 h and a snack around 10:00 h. This led to a more pronounced postprandial TAR, which—though this remains speculative—could further be worsened due to parents providing a relatively large breakfast on schooldays to prevent hypoglycemia during their absence. Lunch timing was also dictated by school schedules, on average resulting in a deeper glucose dip. On OOS days, these patterns were less evident, likely due to breakfast being spread across the morning and lunch occurring at more flexible times.

The TIR during the “school window” (8:30–13:30 h) was numerically lower on school days (74.1%) compared to OOS days (79.0%, not statistically tested). This can be attributed to the timing of the “school window.” This window begins at 8:30 h, coinciding with the peak of post‐prandial hyperglycemia following breakfast, which lowers the TIR during this period. This difference is balanced out when evaluated at the 24‐h level, and is balanced out because the “after school” hours on OOS days exhibit slightly higher average sensor glucose levels.

This study’s findings may help alleviate health care provider and parental concerns about poor glycemic management in children during school days. Consistent good glycemic control is expected to have a positive impact on long‐term diabetes complications, including micro and macrovascular issues, and also positively influence brain development and cognitive function, particularly in children [1, 2]. These results align with other real‐world evidence on the MiniMed 780G system which demonstrates the system and algorithm’s capability to handle changing daily routines, such as managing Ramadan [7], sick days [8], temp target (e.g., due to exercise) [9], and seasonal variations [10].

A key limitation of this study is that CareLink Personal lacks information as well as granularity. For example, age is self‐reported in broad categories, with no further distinction within the <16‐year group. As a result, we may have included users that may not yet attend school, while others may have already completed it. Based on the average total daily insulin dose, we believe that most users in this group are toward the older end of the range. Second, school hours can vary between schools. To account for this, we defined a narrow school window. Although most school days end later in the afternoon, we capped the school window at 13:30 to minimize the influence of non‐school hours on school hours. More importantly, we addressed this limitation by conducting analyses at an hourly level. The clear peak in sensor glucose at breakfast, followed by a dip before lunch, supports that the defined school window accurately captures typical school‐day routines and underscores the validity of results. Third, sick days and absences were not captured in the analyses. However, we believe that such days do not have a strict school routine and have, therefore, attenuated the observed school routine pattern in the hourly analyses. In other words, excluding sick or absent days from the dataset would likely make that pattern even more pronounced. Fourth, we included only days with 100% auto mode use. However, the sensitivity analysis without this criterion showed only minor differences compared to the main analysis, which were not clinically meaningful. Finally, there is the point of external validity to individual children. We acknowledge that these findings are based on group‐level data and may not be generalizable to every individual child at every given time. The strengths of this study include the use of a well‐established methodology, which has been clearly described previously and is frequently applied in similar research, the large sample size, the broad age range of children, and the inclusion of all children in Italy using the system, encompassing a wide range of socioeconomic backgrounds.

In conclusion, this real‐world study demonstrates that Italian CwT1D using the MiniMed 780G system in auto mode on average achieve international glycemic targets on school days as well as OOS days. The system’s ability to autocorrect and dynamically modulate insulin delivery every 5 min allows children to reach glycemic targets regardless of the variability in schedules between school and non‐school days.

## Author Contributions

Jen McVean, Tim van den Heuvel, and Ohad Cohen conceptualized the study. Benedikt Voelker performed the analysis. Tim van den Heuvel drafted the manuscript. All the authors contributed to reviewing and editing the manuscript.

## Funding

No funding was received for this manuscript.

## Disclosure

All the authors have approved the final version of the manuscript, and agree to be accountable for the content and conclusions of the article. All sections of the manuscript underwent language polishing. All AI‐assisted content was reviewed and verified by Tim van den Heuvel as the primary author and by all co‐authors during the review process.

## Conflicts of Interest

Some authors are employees of Medtronic.

## Supporting Information

Additional supporting information can be found online in the Supporting Information section.

## Supporting information


**Supporting Information** Sensitivity analysis without auto mode filter. Data are shown as means ± standard deviation. CV, coefficient of variation; GMI, glucose management indicator; OOS, out‐of‐school; SG, sensor glucose; std, standard deviation; TA180, time above 180; TA250, time above 250; TB54, time below 54; TB70, time below 70; TIR, time in range; TITR, time in tight range.

## Data Availability

All aggregated data have been provided in this manuscript. More granular data are subject to IP (intellectual property) and cannot be shared.
