# Supplementary material for: Achieving Glycemic Targets in and out‐of‐School: Real‐World Data From 1341 Italian Children Using the MiniMed 780G System During Auto Mode
Source: Pediatr Diabetes. 2026 Apr 8;2026:1653728. doi: 10.1155/pedi/1653728 (PMC13060754; doi:10.1155/pedi/1653728)
Supplement: Supplementary file 1 — Supporting Information Sensitivity analysis without auto mode filter. Data are shown as means ± standard deviation. CV, coefficient of variation; GMI, glucose management indicator; OOS, out‐of‐school; SG, sensor glucose; std, standard deviation; TA180, time above 180; TA250, time above 250; TB54, time below 54; TB70, time below 70; TIR, time in range; TITR, time in tight range. [file PEDI-2026-1653728-s001.docx]

Supplemental table: Sensitivity analysis without automode filter

| **​No Automode Filter** | **24 hour window** |  | **School window**  **(8:30-13:30h)** | |
| --- | --- | --- | --- | --- |
| **​** | School Days​ | OOS Days | School Days​ | OOS days |
| **General** |  |  |  |  |
| **Users Count​** | 1676 | 1676 | 1676 | 1676 |
| **System Use, days​** | 151 | 166 | 151 | 166 |
| **Days with 100% automode use (%)** | 137​ (91) | 147​ (89) | 137​ (91) | 147​ (89) |
| **Sensor Use, %​** | 94.8 | 93.5 | 94.8 | 93.5 |
| **Auto Mode Use, %​** | 94.9 | 93.7 | 94.9 | 93.7 |
| **Glycemic metrics** |  |  |  |  |
| **SG mean, mg/dL​** | 153 ± 14.4 | 154 ± 14.7 | 151 ± 18.2 | 142 ± 15.1 |
| **SG std, mg/dL​** | 53.2 ± 10.2 | 55.3 ± 10.6 | 49.8 ±  11.2 | 49.3 ± 11.3 |
| **SG CV, %​** | 34.7 ± 4.68 | 35.7  ± 4.65 | 32.9 ± 5.41 | 34.4 ± 5.53 |
| **GMI, %​** | 6.96 ±  0.34 | 7.00 ± 0.35 | 6.92 ± 0.44 | 6.71 ± 0.36 |
| **TIR, %​** | 71.7 ± 9.20 | 70.6 ± 9.11 | 72.6  ± 12.3 | 77.7 ± 9.99 |
| **TITR, %​** | 46.6±  9.00 | 46.7 ± 8.74 | 47.2 ± 12.9 | 56.8   ±11.5 |
| **TB70, %​** | 2.19 ± 1.69 | 2.00  ± 1.57 | 2.37 ± 2.35 | 2.42 ± 2.27 |
| **TB54, %​** | 0.42 ± 0.48 | 0.38 ± 0.43 | 0.44 ± 0.64 | 0.44 ± 0.60 |
| **TA180, %​** | 26.1 ± 9.39 | 27.4 ± 9.31 | 25.0 ± 12.4 | 19.9 ± 9.87 |
| **TA250, %​** | 6.05 ± 4.78 | 6.96 ± 5.14 | 5.30 ±  5.57 | 4.48 ± 4.33 |

Data are shown as means ± standard deviation/ OOS, out of school/ SG, sensor glucose/ std, standard deviation/ CV, coefficient of variation/ GMI, glucose management indicator/ TIR, time in range/ TITR, time in tight range/ TB70, time below 70/ TB54, time below 54/ TA180, time above 180/ TA250, time above 250
